# Limbic encephalitis – A case report of atypical dementia syndrome with potentially therapeutic consequence

**DOI:** 10.1192/j.eurpsy.2024.1424

**Published:** 2024-08-27

**Authors:** F. Xepapadakos, W. Kawohl

**Affiliations:** ^1^Psychiatry, Clienia Schlössli AG, Oetwil am See/Zurich, Switzerland; ^2^Department of Basic and Clinical Sciences, University of Nicosia Medical School, Nicosia, Cyprus

## Abstract

**Introduction:**

Limbic encephalitis (LE) is a subacute or chronic, non-infectious inflammation of the brain, usually occurring in adulthood, with predominant involvement of mesiotemporal structures and a clinical manifestation consisting mainly of new memory impairment, affective disorder, temporal lobe epilepsy, psychoses, etc.

**Objectives:**

To point out the importance of knowledge of potentially treatable dementia syndromes such as atypical manifestation of probably LE.

**Methods:**

We present a clinical case of a 47-years-old woman with an atypical dementia syndrome and typical radiological findings corresponding to a LE, among others, but without the previously known immunological antibodies.

**Results:**

According to the literature, the diverse subsyndromes of LE can be subsumed under the two main categories of “paraneoplastic” and “non-paraneoplastic”. In addition to the acute and subacute courses, there is increasing evidence for chronic, slowly progressive courses, which expand the spectrum of potentially treatable dementia syndromes. Understanding and knowledge of the broad, clinical syndrome of LE have increased dramatically in recent years. Both nosological classification through differentiated diagnosis and specific therapeutic protocols have become increasingly developed and established. Nevertheless, there are rare clinical cases with a clinical phenotype and radiological findings that correspond to LE, but are both non-paraneoplastic in origin and seronegative with respect to the previously known immunological typing by autoantibodies. This gray area of nosological entity represents a diagnostic and therapeutic challenge.

**Conclusions:**

The authors would like to point out the importance of an adequate diagnosis of the forms of LE that have been nosologically classified so far and are partly well treatable. Limbic encephalitis is an important differential diagnosis in dementia, especially in young patients with atypical courses. There is a need for further research regarding better diagnosis and therapy of the so far immunologically unidentifiable forms of clinical LE.

Literature:

Bazir Ahmad et al., Practical Neurology 2011

Guidelines of the German Neurological Society (DGN), 2008

Leypoldt et al., Akt Neurol 2012

Prüss et al., Neurology 2012

**Disclosure of Interest:**

None Declared

